# Direct Oral Anticoagulants versus Warfarin in Octogenarians with Nonvalvular Atrial Fibrillation: A Systematic Review and Meta-Analysis

**DOI:** 10.3390/jcm10225268

**Published:** 2021-11-12

**Authors:** Clara Bonanad, Sergio García-Blas, Javier Torres Llergo, Rosa Fernández-Olmo, Pablo Díez-Villanueva, Albert Ariza-Solé, Manuel Martínez-Sellés, Sergio Raposeiras, Ana Ayesta, Vicente Bertomeu-González, Francisco Tarazona Santabalbina, Lorenzo Facila, David Vivas, Ana Gabaldón-Pérez, Vicente Bodi, Julio Nuñez, Alberto Cordero

**Affiliations:** 1 Servicio de Cardiología, Hospital Clínico Universitario de Valencia, 46010 Valencia, Spain; clarabonanad@gmail.com (C.B.); sergiogarciablas@gmail.com (S.G.-B.); anagabaldonperez@gmail.com (A.G.-P.); vicentbodi@hotmail.com (V.B.); julio.nunez@uv.es (J.N.); 2 Instituto de Investigación Sanitaria INCLIVA, 46010 Valencia, Spain; 3 Department of Medicine, Medicine Faculty, 46010 Valencia, Spain; 4 Centro de Investigación Biomédica en Red de Enfermedades Cardiovasculares (CIBER-CV), 28029 Madrid, Spain; mmselles@secardiologia.es (M.M.-S.); vbertog@gmail.com (V.B.-G.); 5 Cardiology Department, Hospital Universitario de Jaén, 23007 Jaén, Spain; javiertorresllergo@gmail.com (J.T.L.); mariarosafernandezolmo@gmail.com (R.F.-O.); 6 Cardiology Department, Hospital Universitario de La Princesa, 28006 Madrid, Spain; pablo_diez_villanueva@hotmail.com; 7 Cardiology Department, Hospital Universitari de Bellvitge, 08907 L’Hospitalet de Llobregat, Spain; aariza@bellvitgehospital.cat; 8 Cardiology Department, Hospital Universitario Gregorio Marañón, 28007 Madrid, Spain; 9 Cardiology Department, Hospital Álvaro Cunqueiro, 36213 Vigo, Spain; raposeiras26@hotmail.com; 10 Cardiology Department, Hospital Central de Asturias, 33011 Oviedo, Spain; ana.ayestalopez@gmail.com; 11 Cardiology Department, Hospital Universitario de San Joan, 03550 Alicante, Spain; 12 Department of Clinical Medicine, Universidad Miguel Hernandez, 03550 Alicante, Spain; 13 Geriatrics Department, Hospital Universitario de la Ribera, 46600 Alzira, Spain; fjtarazonas@gmail.com; 14 Cardiology Department, Hospital General Universitario de Valencia, 46014 Valencia, Spain; lorenzofacila@svcardio.org; 15 Instituto Cardiovascular, Hospital Clínico San Carlos, 28040 Madrid, Spain; dvivas@secardiologia.es

**Keywords:** elderly, acute coronary syndrome, myocardial infarction, vitamin-K antagonist, direct oral anticoagulants

## Abstract

Direct oral anticoagulants (DOACs) have been demonstrated to be more effective and safer than vitamin-K antagonist (VKA) for stroke prevention in patients with nonvalvular atrial fibrillation (AF). This meta-analysis aims to assess the effect of DOACS vs. VKA in patients ≥ 80 and AF. Primary endpoints were stroke or systemic embolism and all-cause death. Secondary endpoints included major bleeding, intracranial bleeding, and gastrointestinal bleeding. A random-effects model was selected due to significant heterogeneity. A total of 147,067 patients from 16 studies were included, 71,913 (48.90%) treated with DOACs and 75,154 with VKA (51.10%). The stroke rate was significantly lower in DOACs group compared with warfarin group (Relative risk (RR): 0.72; 95% confidence interval (CI): 0.63–0.82; *p* < 0.001). All-cause mortality was significantly lower in DOACs group compared with warfarin group (RR: 0.82; 95% CI: 0.70–0.96; *p* = 0.012). Compared to warfarin, DOACs were not associated with reductions in major bleeding (RR: 0.85, 95% CI 0.69–1.04; *p* = 0.108) or gastrointestinal bleeding risk (RR: 1.08, 95% CI 0.76–1.53; *p* = 0.678) but a 43% reduction of intracranial bleeding (RR: 0.47, IC 95% 0.36–0.60; *p* < 0.001) was observed. Our meta-analysis demonstrates that DOACs are effective and safe with statistical superiority when compared with warfarin in octogenarians with AF.

## 1. Introduction

The incidence of nonvalvular atrial fibrillation (AF) increases exponentially with age [1,2], and its prevalence varies from 0.1% among patients younger than 55 years to 9–17.7% in those aged 80 years or older [2,3,4,5,6]. Octogenarians are growing faster than those above age 65, and the global population aged 80 years and over is projected to triple in 2050 [7].

AF is an independent risk factor for stroke, and age per se entails a greater risk of strokes [8]. Furthermore, age is considered a risk factor for CHA2DS2VASc and HASBLED scores [9]. Besides, elderly patients commonly present multiple comorbidities such as dementia, high risk of falls, chronic kidney disease, hypertension, and diabetes, which may lead to greater complexity in their management [10].

Direct oral anticoagulants (DOACs) have been demonstrated to be more effective and safer than vitamin K antagonists (VKA) for stroke prevention in patients with nonvalvular AF [11,12,13,14]. Moreover, DOACs overcome the limitations of VKA, showing fewer drug and dietary interactions and rapid onset of action. Based on these advantages over VKA, DOACs have been recommended as the first-choice therapy to prevent stroke in AF [15,16,17]. However, the potential benefits of DOACs have not been specifically studied in octogenarians with AF because the elderly population was underrepresented in the pivotal randomized controlled trials (RCTs), which excluded these high-risk patients. The rate of patients aged ≥80 and ≥85 years in the pivotal RCTs was 13–17% and 4–7%, respectively [18,19,20,21,22,23].

Considering the current lack of information from clinical trials to make clinical recommendations about the efficacy of oral anticoagulants in the management of patients aged 80 or older with AF, and to provide some insights into this issue, we conducted a meta-analysis to evaluate the clinical benefit of DOACs compared to VKA in octogenarians with nonvalvular AF.

## 2. Material and Methods

We performed an intention-to-treat meta-analysis in line with recommendations from the Cochrane Collaboration and the Preferred Reporting Items for Systematic Reviews and Meta-Analyses (PRISMA) Statement [24] using standard software (STATA 14.3, STATA Corp, College Station, TX, USA). We included studies that compared DOACs and VKA for patients with nonvalvular AF with an indication for stroke prevention. The primary endpoints analyzed were stroke and systemic embolism, and all-cause death. According to each study’s definitions, secondary endpoints were major bleeding, intracranial bleeding, and gastrointestinal (GI) bleeding. The raw numbers of incident endpoints reported in each study were used.

**Figure 1:**
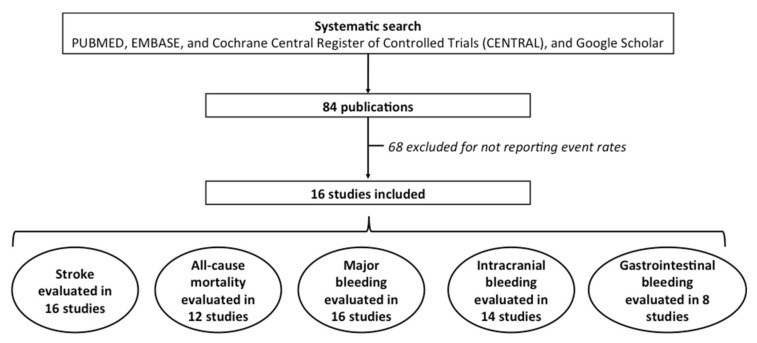
PRISMA flow diagram.

We conducted a systematic search (using PUBMED, EMBASE, and Cochrane Central Register of Controlled Trials (CENTRAL), and Google Scholar), without language restriction, for studies and randomized clinical trials using the Medical Subject Headings terms “dabigatran”, “rivaroxaban”, “apixaban”, “edoxaban”, “warfarin”, “vitamin K antagonist”, “octogenarians”, “elderly”, or “very elderly”. The systematic search was restricted from 2010 to 2020. Information on sample size, treatment type and outcomes were collected from the published papers. For the eligible studies, two authors independently abstracted data into a standardized database; discrepancies in data extraction and quality assessment were resolved by discussion or consensus with a third author. For the studies that evaluated different doses or different drugs each strategy was analyzed as a separate study (Figure 1).

### Data Synthesis and Statistical Analyses

The percentage of variability across studies attributable to heterogeneity beyond chance was estimated using the I2 statistic. A random-effects model was selected because significant heterogeneity was observed. Sensitivity analyses were performed by the analysis of between-study variation using the study-specific standard errors. Potential sources of heterogeneity between trials or treatments were tested by meta-regression analyses and the effect of age, the number of patients treated, type of study (randomization) or propensity score matching, gender distribution, the prevalence of hypertension, diabetes, previous stroke, or heart failure was assessed. We also tested the small-study effect by the Egger test [25].

## 3. Results

The summary of the 16 studies is presented in Table 1. A total of 147,067 patients were included: 71,913 (48.90%) treated with DOACs and 75,154 (51.10%) with VKA. Inclusion criteria for the study were age ≥ 80 in 13 studies (*n* = 143,831 patients), ≥85 in two (*n* = 1490) and ≥90 in one (*n* = 1746). Mean age was 86.2 (SD 2.6) years. A total of 34,448 (47.90%) patients received rivaroxaban, 20,295 (28.22%) apixaban, 14,641 (20.36%) dabigatran, 492 (0.69%) edoxaban and; 2037 (2.83%) a non-specified DOAC.

**Table 1:** Summary of the studies included in the analyses.

Study	Study Type	First Author	Year	Country	Age Criteria	Follow-Up (Days)	Adjustment	Treatment	Control	Treatment (*n*)	Control (*n*)	Mean Age	Males
ARISTOTLE [19]	RCT	Halvorsen	2014	World-wide	≥80	900	Randomization	Apixaban	VKA	1218	1218	82	512
AVERROES [20]	RCT	Ng	2016	World-wide	≥85	375	Randomization	Apixaban	Aspirin	180	186		163
CARDIOCHUS [26]	O	Rodriguez-Mañero	2017	Spain	≥80	696	Multivariate analyses	DOACs	VKA	220	2976	84.6	1600
RELY [27]	RCT	Lauw	2017	Word-wide	≥80	730	Randomization	Dabigatran 110	VKA	1009	1009	83	1678
RELY [27]	RCT	Lauw		Word-wide	≥80	730	Randomization	Dabigatran 150	VKA	1009	1009	83	1678
National Health Insurance Research Database [28]	O	Chao	2018	Taiwan	≥90	670	Propensity score matching	DOACs	VKA	978	768	92.4	778
ARISTOPHANES [8]	O	Deitelzweig	2019	US	≥80	243	Propensity score matching	Apixaban	VKA	18,897	18,897	85.3	5383
ARISTOPHANES [8]	O	Deitelzweig	2019	US	≥80	255	Propensity score matching	Dabigatran	VKA	6698	6698	84.4	15,069
ARISTOPHANES [8]	O	Deitelzweig	2019	US	≥80	255	Propensity score matching	Rivaroxaban	VKA	25,917	25,917	85.1	5655
System National des Données de Sante- Rivaroxaban	O	Blin [29]	2019	France	≥80	365	Propensity score matching	Rivaroxaban	VKA	7536	7536	88.5	5482
	O	Blin [30]	2019	France	≥80	365	Propensity score matching	Dabigatran	VKA	5925	5925	88.2	4932
STAST2- REGISTER [31]	O	Poli	2019	Italy	≥85	400	Multivariate analyses	DOACs	VKA	464	660	87.9	352
ELDERCARE-AF [18]	RCT	Okumura	2020	Japan	≥80	466	Randomization	Edoxaban 15	Placebo	492	492	86.6	419
FANTASIIA [32]	O	Anguita-Sanchez	2020	Spain	≥80	1095	Multivariate analyses	DOACs	VKA	123	451	83.9	254
Atrial Fibrillation Research Database [33]	O	Russo	2020	Italy	≥80	855	Propensity score matching	DOACs	VKA	252	504	84.5	329
SAFIR [34]	O	Hanon	2021	France	≥80	286	Propensity score matching	Rivaroxaban	VKA	995	908	86	692

Abbreviations: RCT: Randomized Clinical Trial; O: Observational. The stroke rate was significantly lower in DOACs group compared with warfarin group [Relative risk (RR): 0.72; 95% confidence interval (CI): 0.61–0.84; *p* < 0.001] (Figure 2). All-cause mortality could be assessed in 12 studies and was significantly lower in DOACs group compared with warfarin group [RR: 0.82; 95% CI: 0.70–0.96; *p* = 0.012] (Figure 3).

**Figure 2:**
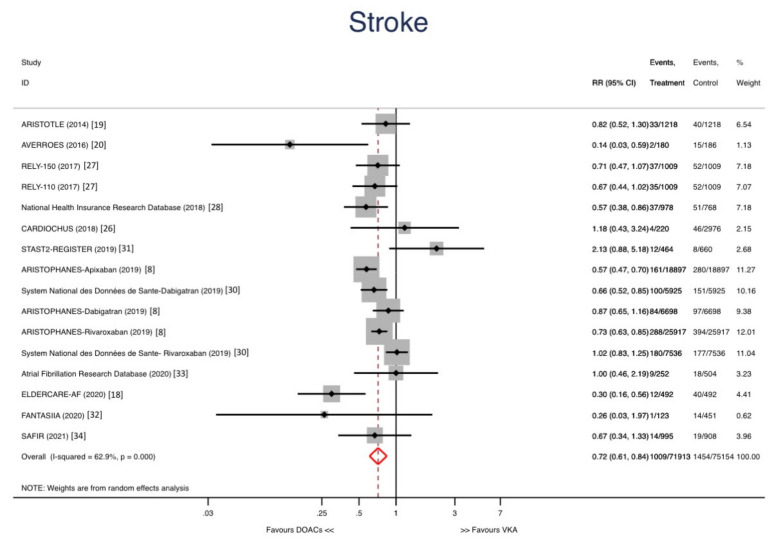
Forest plots showing the pooled risk ratio (HR) with 95% confidence intervals for stroke.

**Figure 3:**
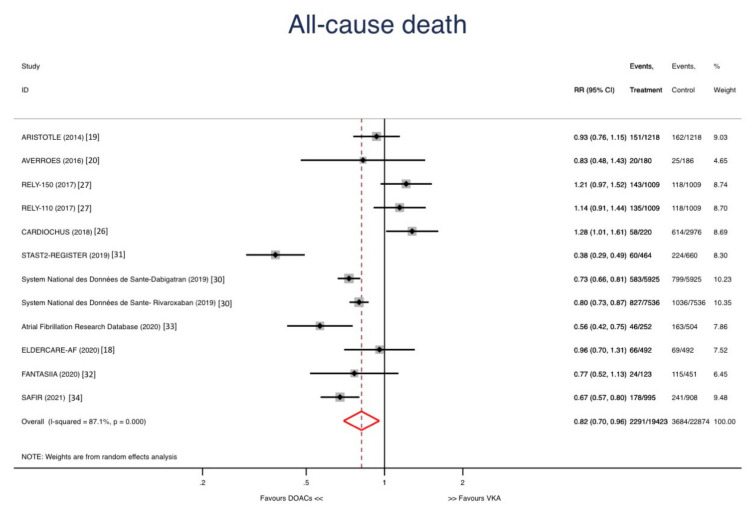
Forest plots showing the pooled hazard ratio (HR) with 95% confidence intervals for all-cause death.

**Figure 4:**
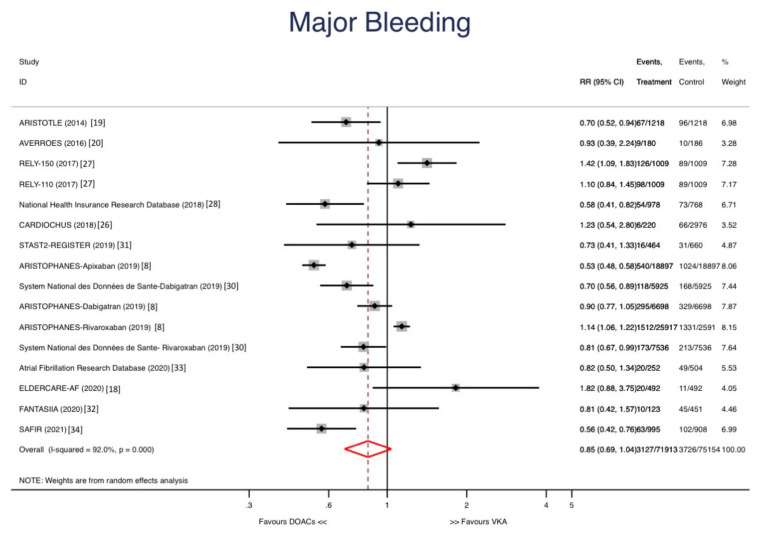
Forest plots showing the pooled hazard ratio (HR) with 95% confidence intervals for major bleeding.

**Figure 5:**
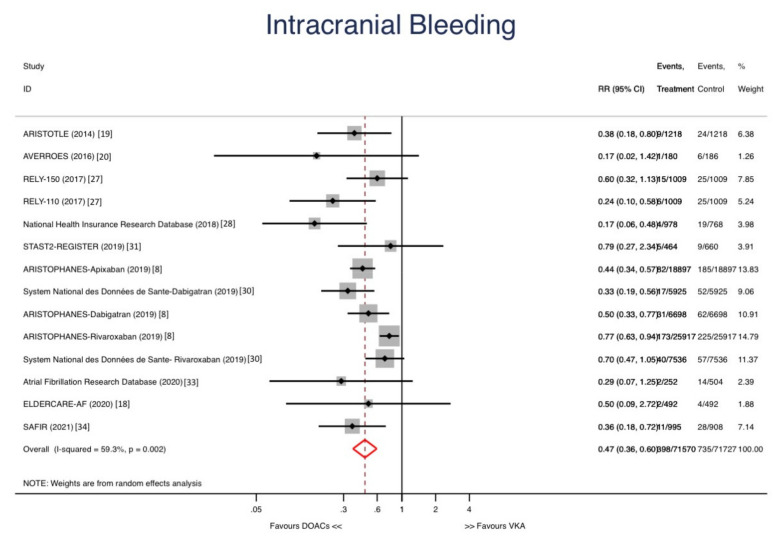
Forest plots showing the pooled hazard ratio (HR) with 95% confidence intervals for intracranial bleeding.

DOACs were not associated with reductions in major bleeding (RR: 0.85, 95% CI 0.69–1.04; *p* = 0.108) (Figure 4) but reduced the risk of intracranial bleeding (RR: 0.47, IC 95% 0.36–0.60; *p* < 0.001) (Figure 5). The risk of GI bleeding was similar in both groups (RR: 1.08, 95% CI 0.76–1.53; *p* = 0.678).

### Sensitivity Analyses and Metaregression

**Figure 6:**
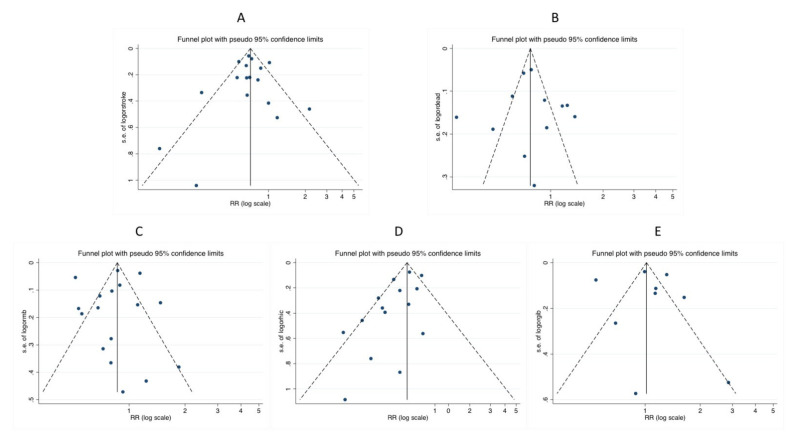
Funnel plot for the primary (**A**) stroke, (**B**) all-cause death, (**C**) major bleeding, (**D**) intra-cranial bleeding and (**E**) gastrointestinal bleeding.

Significant heterogeneity was observed in all endpoints. The funnel plots are presented in Figure 6. Metaregression identified inclusion site in North-America (*p* < 0.001), the ELDERCARE-AF results (*p* = 0.023), control arm different than VKA (*p* = 0.006), and the prevalence of hypertension (*p* = 0.042) as main sources of heterogeneity for stroke risk reduction. The type of DOAC was the main source of sources of heterogeneity for all-cause mortality (*p* < 0.001) and major bleeding (*p* = 0.03) risk reduction. None of the clinical characteristics presented in Table 1 were detected as a source of heterogeneity for gastrointestinal bleeding. No small-study effect was detected for stroke, all-cause mortality, major bleeding, or gastrointestinal bleeding.

Hypertension (*p* = 0.04) was detected as the leading source of heterogenicity for intracranial bleeding, and a small study effect was also detected (Harbor test *p* = 0.029).

## 4. Discussion

This meta-analysis of currently available evidence from the subanalysis of clinical trials and real-world cohorts endorsed the efficacy and safety of DOACS vs. VKA in octogenarians with AF, associating significant reductions in stroke, all-cause mortality, and intracranial bleeding. Prevalence rates of AF increase significantly with age, ranging from 0.1% among patients younger than 55 years to 9–17.7% in those aged 80 years or older [2,3,4,5,6]. Due to the increased life expectancy, the number of patients aged 80 years or older with AF is expected to rise 4-fold by 2050, representing more than half of all AF cases [2].

Patients with AF aged 75 years or older have a worse prognosis than those between 65 and 74 years, with higher mortality rates and major adverse cardiac events [35]. The classic BAFTA study carried out with warfarin, and the more recent PREFER-AF registry has shown the net clinical benefit of anticoagulation among the elderly or very old population (>85 years) in terms of survival and reduction of thromboembolic events [36,37]. The advent of DOACS has improved prescription rates of oral anticoagulation (OAC) in older people, but OAC remains underutilized in up to 30% of patients with high stroke risk [38]. Every decade increase in age is related to up to a 14% reduction in VKA use, regardless of other stroke risk factors [39]. It has been described that antiplatelet therapy, age over 90 years, falls risk, and nursing home residency are the main reasons for not starting OAC in frail elderly, despite a clear indication that frailty is associated with an increased risk of stroke but not with the risk of major bleeding [40].

There are several important findings in this meta-analysis evaluating the clinical benefit of DOACs in octogenarians with AF. First, compared to VKA, DOACs significantly reduced the risk of stroke, all-cause mortality, and intracranial bleeding in patients with AF aged 80 or older. Moreover, in this group of patients, treatment with DOACs was not associated with greater major or gastrointestinal bleeding risk than VKA. Therefore, old age alone should not be a criterion for withholding OAC therapy with DOACs. Nonetheless, these results do not contradict the fact that the use of OAC in octogenarian patients with AF should be based on an individualized, case-by-case approach with an adequate assessment of stroke risk, bleeding risk, and comorbidities [3].

Our results take a step forward within the currently available evidence, especially because although different meta-analyses analyzed the efficacy and safety of the use of DOACs in elderly patients [3,41,42,43,44,45,46], there is currently no one that has focused on patients over 80 years of age. Furthermore, a dedicated trial with a head-to-head comparison among DOACs in this group of patients is unlikely. This lack of evidence in octogenarians justifies our meta-analysis that demonstrated the beneficial effect of DOACs on stroke and all-cause mortality. The highest reduction associated with DOACs were for intracranial hemorrhage, a devastating bleeding complication that is reduced by 43%. Octogenarians have a high risk of both bleeding and stroke, which could explain the absence of difference in major bleeding rates in patients treated with DOACs or VKA. Nonetheless, the significant reductions of stroke, all-cause mortality, and intracranial bleeding highlight the net benefit of DOACs vs. VKA in octogenarians with AF.

### Limitations of the Study

This meta-analysis might be limited because it mainly included a subanalysis of randomized clinical trials and observational studies and, therefore, evidence might be less reliable than randomized trials. Nonetheless, this also highlights the unmet need for accurately designed and powered clinical trials in elderly patients with AF. We also observed differences in clinical characteristics and meant follow-up in the studies included, which might explain the significant heterogeneity observed in all the endpoints. In the review of the manuscripts we have not found information about treatment crossover between both arms, a significant percentage of crossover could affect the quality of the results. In the AVERROES [20] study, control patients took aspirin while control patients in the ELDERCARE-AF [18] were under placebo. Finally, there is a limitation regarding the analysis of the geriatric profile in the elderly, taking into account that RCTs, based on ethical and practical considerations, usually do not include patients with complex real-life conditions (as frailty, multimorbidity, and polypharmacy) and that there are currently no unified recommendations about the best dose for DOACs in this group of patients.

## 5. Conclusions

This meta-analysis of more than 147,000 patients with age >80 and AF demonstrates the superiority of DOACs compared to VKA in stroke prevention and all-cause mortality and intracranial bleeding.

## Data Availability

Data are available from the authors upon reasonable request.
